# Thromboxane A2 receptor antagonist SQ29548 reduces ischemic stroke-induced microglia/macrophages activation and enrichment, and ameliorates brain injury

**DOI:** 10.1038/srep35885

**Published:** 2016-10-24

**Authors:** Aijuan Yan, Tingting Zhang, Xiao Yang, Jiaxiang Shao, Ningzhen Fu, Fanxia Shen, Yi Fu, Weiliang Xia

**Affiliations:** 1 Department of Neurology & Institute of Neurology, Rui Jin Hospital, Shanghai Jiao Tong University, 200025, China; 2 School of Biomedical Engineering & Med-X Research Institute, Shanghai Jiao Tong University, Shanghai, 200030, China; 3 Zhiyuan College, Shanghai Jiao Tong University, Shanghai, 200240, China

## Abstract

Thromboxane A2 receptor (TXA2R) activation is thought to be involved in thrombosis/hemostasis and inflammation responses. We have previously shown that TXA2R antagonist SQ29548 attenuates BV2 microglia activation by suppression of ERK pathway, but its effect is not tested *in vivo*. The present study aims to explore the role of TXA2R on microglia/macrophages activation after ischemia/reperfusion brain injury in mice. Adult male ICR mice underwent 90-min transient middle cerebral artery occlusion (tMCAO). Immediately and 24 h after reperfusion, SQ29548 was administered twice to the ipsilateral ventricle (10 μl, 2.6 μmol/ml, per dose). Cerebral infarction volume, inflammatory cytokines release and microglia/macrophages activation were measured using the cresyl violet method, quantitative polymerase chain reaction (qPCR), and immunofluorescence double staining, respectively. Expression of TXA2R was significantly increased in the ipsilateral brain tissue after ischemia/reperfusion, which was also found to co-localize with activated microglia/macrophages in the infarct area. Administration of SQ29548 inhibited microglia/macrophages activation and enrichment, including both M1 and M2 phenotypes, and attenuated ischemia-induced IL-1ß, IL-6, and TNF-α up-regulation and iNOS release. TXA2R antagonist SQ29548 inhibited ischemia-induced inflammatory response and furthermore reduced microglia/macrophages activation and ischemic/reperfusion brain injury.

Cerebral infarction is one of the leading causes of death and permanent disability worldwide^1^. The pathophysiological mechanisms, which include inflammation, apoptosis, excitotoxicity and peri-infarct depolarization, are extraordinarily complex^2^. However, effective therapeutic methods for the treatment of ischemic stroke are limited and new methods are needed. Microglia, similar to peripheral macrophages, are the resident immune cells of the central nervous system and respond to micro-environmental changes. Ischemic stroke can lead to microglia activation and macrophages infiltration. Neuroinflammation mediated by activation of microglia/macrophages is an important factor that contributes to neuron death and infarct area diffusion in ischemia/reperfusion injury^3^,^4^. A large body of evidence demonstrated that inhibition of microglia/macrophages activation and enrichment after cerebral infarction can improve neurological outcome and ameliorate brain injury^5^,^6^,^7^.

The thromboxane A2 receptor (TXA2R) is a seven-transmembrane G-protein-coupled receptor localized both on the cell membrane and in intracellular compartments^8^. TXA2R represents an established target for antithrombotic therapies, since it is mainly expressed on circulating platelets, where its activation by the thromboxane A2 (TXA2) mediates platelet activation and aggregation. Recently, however, it is clear that TXA2R exhibits a wide distribution in different cell types and different organ systems, and new functions of TXA2R have been assigned in peripheral nervous system, kidney, allergy and inflammation, immunity, angiogenesis and metastasis of cancer cell^8^. TXA2R activation is thought to be involved in thrombosis/hemostasis, inflammation, modulation of immune responses^9^. Sobolesky and coworkers found that in urothelial cancer TXA2R signaling played a role in the regulation of FOXO3 activity, mediated in part through phosphorylation and deacetylation^10^. Expression of TXA2R was elevated in rat cerebral arteries and microvessels after subarachnoid hemorrhage^11^. A recent study also proposed that TXA2R antagonism was a new concept in atherothrombosis and stroke prevention^12^, which is evidenced in our previous study where a polymorphism of TXA2R was found to associate with cerebral infarction in a Chinese Han population^13^,^14^. Our group has recently discovered that TXA2R agonist U46619 could activate BV2 microglia to release inflammatory cytokines, and TXA2R antagonist SQ29548 could repress the process and reduce inflammation cytokines release^15^. Clinically, thromboxane biosynthesis was increased in patients with cerebral infarction^16^,^17^. Collectively, these studies point to a role of TXA2R in neuroimmune modulation after stroke and we thus hypothesize that TXA2R participates in the processes of ischemia/reperfusion-induced microglia/macrophages activation and enrichment.

The function of TXA2R antagonists in ischemia/reperfusion-induced microglia/macrophages activation and enrichment is unknown. Microglia/macrophages are the main target of the inflammatory response, thus our study was undertaken to: (1) elucidate the expression changes of TXA2R in a mouse model of ischemic/reperfusion brain injury (transient middle cerebral artery occlusion, tMCAO); (2) assess the function of TXA2R antagonist to microglia/macrophages activation and enrichment, inflammatory response, and the damage after tMCAO.

## Results

### TXA2R in microglia/macrophages was upregulated in the ipsilateral striatum after tMCAO

**Figure 1:**
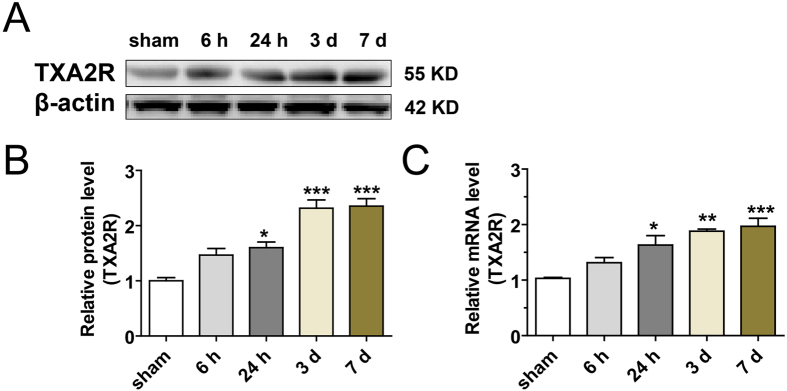
Upregulation of TXA2R in the ipsilateral striatum after ischemia/reperfusion injury. (**A,B**) TXA2R protein expression in the ipsilateral striatum at indicated time after ischemia/reperfusion injury. Full-length blots/gels are presented in Supplementary Figure S1 (n = 4 each time point). (**C**) TXA2R mRNA expression in the ipsilateral striatum at indicated time after ischemia/reperfusion injury (n = 4 each time point).

We first analyzed the time-dependent expression of TXA2R in the ipsilateral striatum and cortex. Western blotting analysis revealed that TXA2R level started to increase at 6 h, with a significant increase at 24 h that lasted up to 7 d after ischemia/reperfusion in the ipsilateral striatum compared to the sham-operated group (Fig. 1A). The level of TXA2R protein from the ipsilateral cortex did not increase compared to sham-operated group at 24 h after tMCAO (Supplementary Figure S1). Consistent with the protein level, mRNA expression of TXA2R in the striatum of the ipsilateral hemisphere began to increase at 6 h and was maintained until 7 d post-injury (Fig. 1C). These data suggested that TXA2R mRNA and protein levels in the ipsilateral hemisphere (mainly striatum) were upregulated after tMCAO.

**Figure 2:**
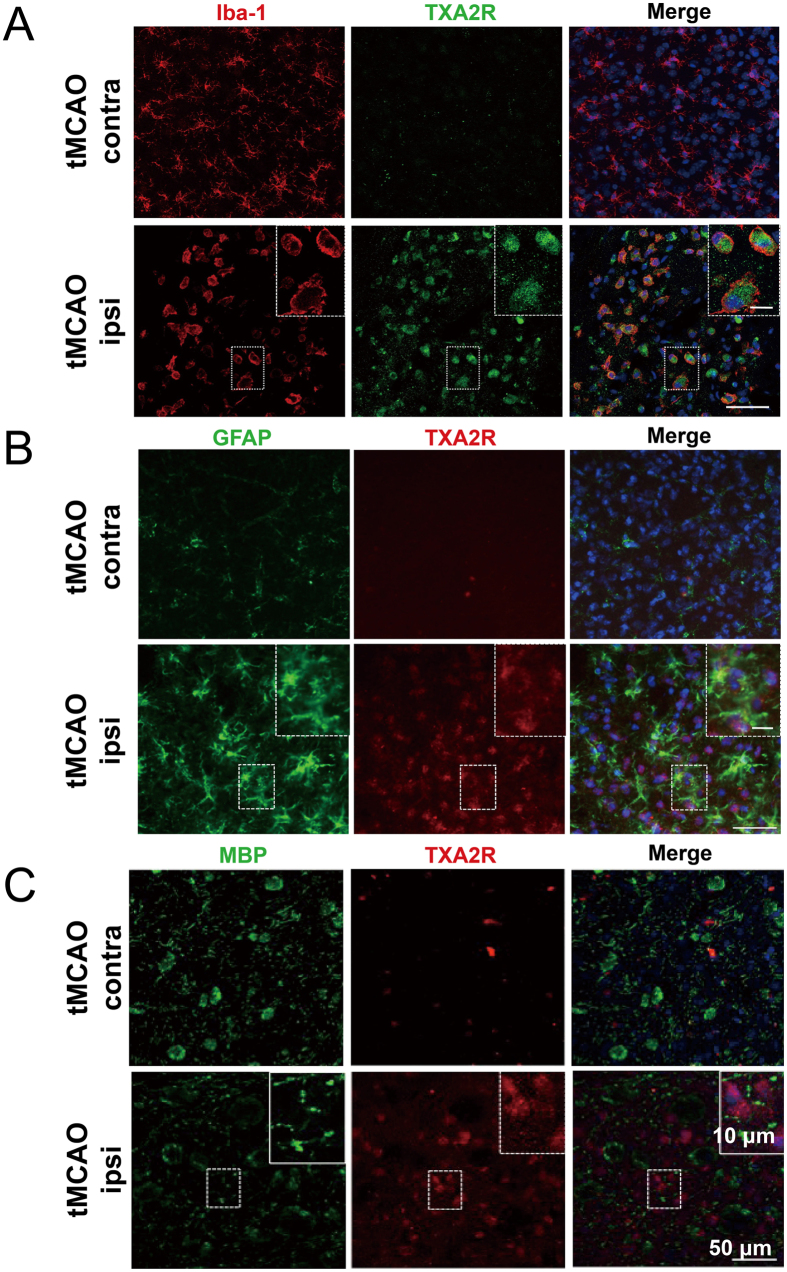
Upregulation of TXA2R expression in microglia/macrophages in tMCAO mice. (**A**) Co-staining of TXA2R (green), Iba-1 (red) and nuclei (blue) in mice brain at 3 d after tMCAO injury. (**B**) Co-staining of TXA2R (red), GFAP (green) and nuclei (blue) in mice brain 3 d after ischemia/reperfusion injury. (**C**) Co-staining of TXA2R (red), MBP (green) and nuclei (blue) in mice brain 3 d after ischemia/reperfusion injury. n = 4, Scale bar = 50 μm.

We next investigated the cell type-specific expression pattern of TXA2R following tMCAO. The above results showed that TXA2R protein level reached a peak at 3 d in the ipsilateral hemisphere following tMCAO. We then used an immunofluorescence double-staining method to determine whether TXA2R was expressed in microglia/macrophages (Iba-1-positive), astrocytes (GFAP-positive) and oligodendrocytes (MBP-positive) at 3 d after reperfusion. TXA2R was highly expressed in activated microglia/macrophages in the ipsilateral striatum compared with the contralateral striatum of the tMCAO group (Fig. 2A). We also detected TXA2R expression in astrocytes and oligodendrocytes, but only a few double-labeled cells were observed in ipsilateral striatum (Fig. 2B,C). These results showed that ischemia/reperfusion induced robust TXA2R expression in microglia/macrophages, but minimal expression in astrocytes and oligodendrocytes.

### SQ29548 inhibited microglia/macrophages activation and enrichment in the infarcted striatum after tMCAO

**Figure 3:**
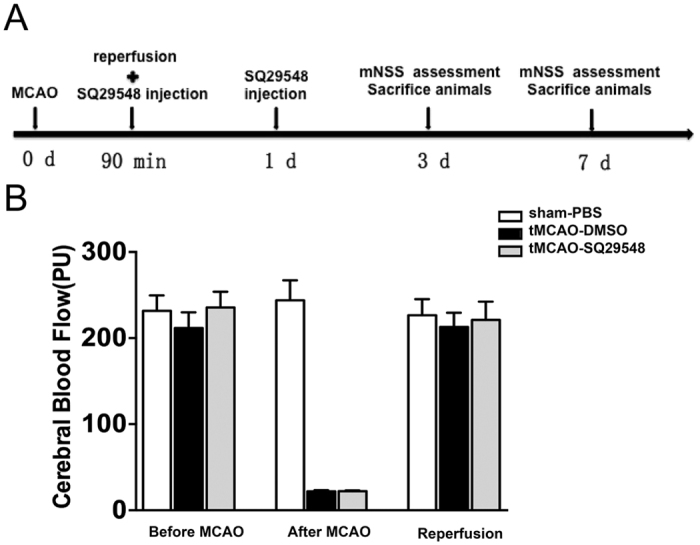
Experimental design and cerebral blood flow during MCAO. (**A**) This diagram illustrated the experimental design including tMCAO, SQ29548 administration, neurobehavioral assessments and animal sacrifice. (**B**) Representation of cerebral blood flow before MCAO (as a baseline), after MCAO, and reperfusion. Sham-PBS n = 8, tMCAO-SQ29548 n = 16, tMCAO-DMSO n = 16.

**Figure 4:**
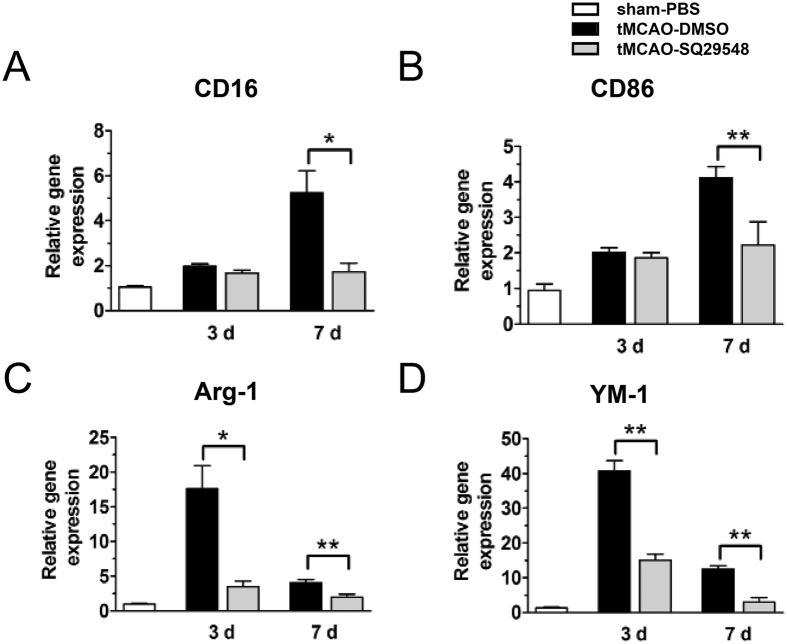
SQ29548 inhibits microglia/macrophages activation after tMCAO injury. mRNA levels of M1 markers CD16 (**A**) and CD86 (**B**) and M2 markers Arg-1 (**C**) and Ym-1 (**D**) in the ipsilateral striatum of sham-PBS (n = 4), tMCAO-DMSO (n = 8) and tMCAO-SQ29548 (n = 8) groups at 3 d, 7 d following tMCAO. Values are the mean ± SEM. *P < 0.05, **P < 0.01.

**Figure 5:**
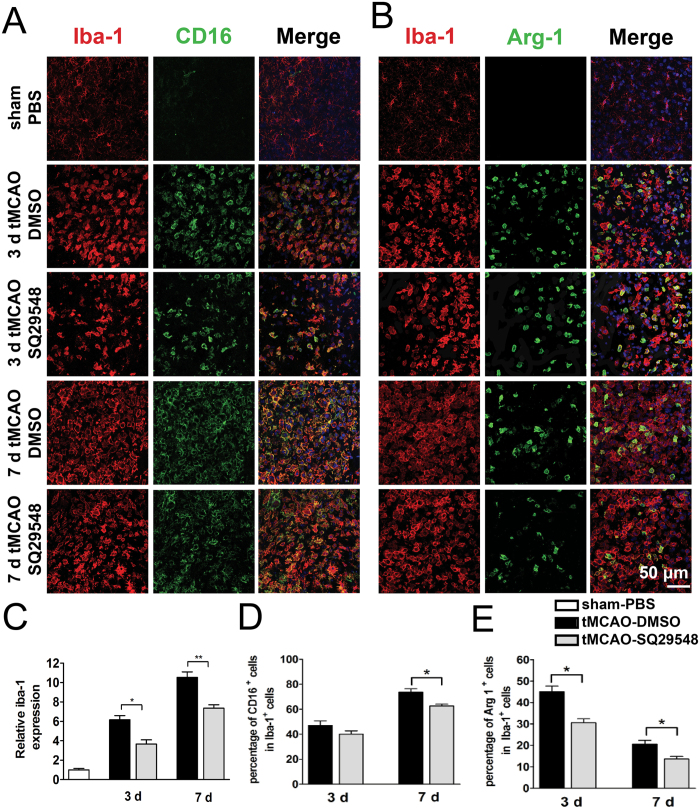
SQ29548 reduces M1/M2-type cells after tMCAO injury. (**A,B**) Immunofluorescence double staining for Iba-1 (red) with CD16 (green) or Arg-1 (green) in the inner boundary of the infarct striatum of sham-PBS (n = 4), tMCAO-DMSO (n = 8) and tMCAO-SQ29548 (n = 8) groups at 3 d, 7 d following tMCAO. (**C**) The quantity of microglia/macrophages in the ischemic cerebral striatum is quantified by the intensity of Iba-1^+^ immunofluorescence. Data were normalized against Iba-1^+^ level of the sham-PBS group. (**D**,**E**) Quantification of the percentage of CD16^+^/Iba-1^+^ and Arg-1^+^/Iba-1^+^ cells. Scale bar = 50 μm. Values are the mean ± SEM. *P < 0.05, **P < 0.01.

Ischemic stroke-induced activation of microglia and infiltration of macrophages contribute to the brain injury after cerebral infarction^18^,^19^. Activated microglia/macrophages include two phenotypes, the classically activated M1 type and the alternatively activated M2 type^20^. Our data showed TXA2R was highly expressed in activated microglia/macrophages in the ipsilateral striatum. Our previous study also discovered that SQ29548 as a highly selective TXA2R antagonist could repress BV2 microglia activation and reduce pro-inflammatory cytokines release^15^. To further understand the impact of TXA2R inhibition on the activation of microglia/macrophages after tMCAO, intracerebroventricular administration of SQ29548 was performed right after reperfusion with a repeated dose 24 h thereafter. The experimental protocol is illustrated in Fig. 3A. Animals with successful ischemia/reperfusion were included for further characterizations when the cerebral blood flow decreased to 20% of the baseline and returned to 80% after suture withdrawal (Fig. 3B). We then analyzed microglia/macrophages activation after tMCAO by quantitative PCR and immunofluorescence double staining. SQ29548 inhibited mRNA expression of M2 markers (Arg-1, Ym-1) (Fig. 4C,D) and M1 markers (CD16, CD86) at 3 d and 7 d after tMCAO (Fig. 4A,B). Some M1- and M2-type genes are also expressed in other cells of the central nervous system or infiltrating immune cells; thus, the results of quantitative PCR reflect changes in these genes in mixed cell types. To determine the changes in microglia/macrophages in the SQ29548-treated group after cerebral ischemia, representative M1- or M2- associated markers were analyzed with the microglia/macrophages marker Iba-1 in the inner boundary of infarction by immunofluorescence double staining. Consistent with the quantitative PCR results, the expression of the M2 marker Arg-1 was low in Iba-1^+^ microglia/macrophages in SQ29548-treated group compared to the DMSO-treated group at 3 d and 7 d after tMCAO (Fig. 5B,E). Similarly the expression of the M1 marker CD16 was lower in Iba-1^+^ cells in the SQ29548-treated group at 7 d after tMCAO (Fig. 5A,D). Activated microglia/macrophages that included M1 and M2 phenotypes were reduced in tMCAO-SQ29548 group. The percentage of Iba-1^+^ microglia/macrophages in tMCAO-SQ29548 group was also obviously lower than that in tMCAO-DMSO group (Fig. 5C). This means that blockage of TXA2R after tMCAO inhibited resident microglia/macrophages to activate and enrich. It is reported that DMSO displayed certain degree of neuroprotective effects, but possibly through a pathway that is unlikely to involve microglia. Katherine R. Leaver’s study demonstrated 10% DMSO and 40% DMSO had no effects on microglia in a rat model of excitotoxic injury^21^. In our control experiment, intracerebroventricular administration of 10% DMSO after tMCAO showed no effects on microglia/macrophages activation as evaluated by M1/M2 marker expression, in comparison to tMCAO mice (Supplementary Figure S3). We also found activated astrocyte were reduced in tMCAO-SQ29548 group than in tMCAO-DMSO group at 3 d after tMCAO (Supplementary Figure 5). The reduction of oligodendrocytes was also attenuated in tMCAO-SQ29548 group (Supplementary Figure 6). Our results suggested that SQ29548 inhibited cerebral ischemia-induced microglia/macrophages activation and enrichment in the brain after tMCAO. Astrocytes and oligodendrocytes are also affected by the application of SQ29548 after ischemic stroke.

### SQ29548 ameliorated inflammatory responses and reduced oxidative stress after tMCAO

**Figure 6:**
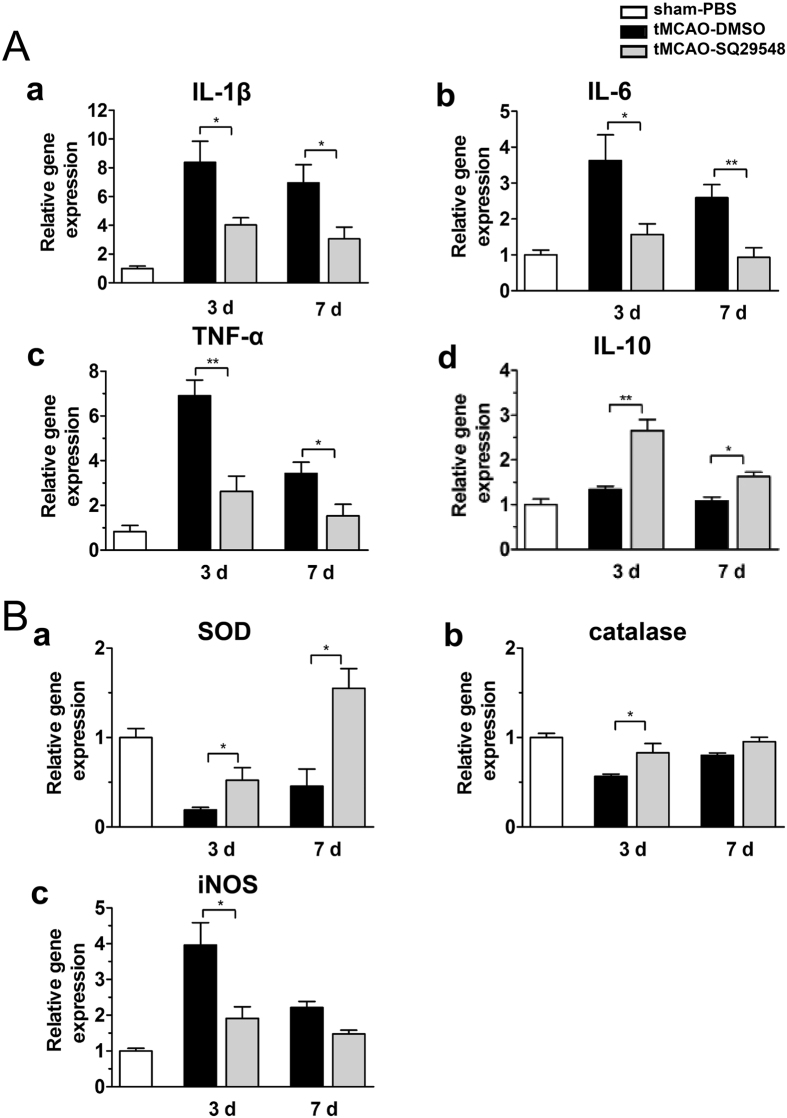
SQ29548 reduced inflammatory cytokine production and alleviated oxidative stress response in tMCAO mice. (**A**) mRNA levels of pro-inflammatory mediators IL-1ß (a), IL-6 (b), TNF-α (c), IL-10 (d) normalized to Rplp0 in the ipsilateral striatum. (**B**) mRNA levels of antioxidant mediator of SOD2 (a), catalase (b) and oxidative stress factor iNOS (c) normalized to Rplp0 in the ipsilateral striatum of sham-PBS (n = 4), tMCAO-DMSO (n = 8) and tMCAO-SQ29548 (n = 8) groups at 3 d, 7 d following tMCAO. Values are mean ± SEM. *P < 0.05, **P < 0.01.

After ischemia, resident microglia are activated and peripheral macrophages are rapidly recruited to the infarct area. Activated microglia/macrophages are mostly related to inducing inflammatory response and recruitment of immune cells after ischemic stroke^19^. Astrocytes and oligodendrocytes are involved in a number of activities during ischemia, including inflammatory response, maintenance of the blood–brain barrier. These glia cells become activated and release pro-inflammatory mediators during the subacute period after ischemic stroke^22^ and respond to injury in a highly localized manner. Previous data showed that SQ29548 inhibited cerebral ischemia-induced microglia/macrophages and astrocytes activation. We then evaluated the anti-inflammatory effect of SQ29548 in ischemic stroke injury. The mRNA levels of IL-1ß, IL-6, and TNF-α were dramatically decreased in the SQ29548-treated group compared with the DMSO-treated group at 3 d and 7 d after tMCAO (Fig. 6Aa–c). IL-10 is regarded as a beneficial cytokine in the immune response^23^. The expression of IL-10 increased by 2-fold at 3 d and by 1.5-fold at 7 d in the SQ29548-treated group compared to the DMSO-treated group after tMCAO (Fig. 6Ad). These effects were unlikely mediated by the vehicle DMSO: intracerebroventricular administration of 10% DMSO after tMCAO had no effects on the mRNA expression of IL-1ß, IL-6, and TNF-α, as compared to tMCAO mice (Supplementary Figure S4A–C). Our results revealed that administration of SQ29548 after tMCAO led to a marked reduction in the expressions of inflammatory cytokines, demonstrating the anti-inflammatory property of SQ29548 for ischemia/reperfusion injury.

Since ROS also plays an important role in cerebral ischemia/reperfusion injury, we continued to investigate the effect of SQ29548 on resistance to oxidative stress. Quantitative PCR assays showed that compared with the DMSO groups, the expression of antioxidant gene of SOD2 (Fig. 6Ba) and catalase (Fig. 6Bb) mRNA levels were apparently increased in SQ29548 group after ischemia/reperfusion injury. And, iNOS mRNA level was significantly decreased in the SQ29548 treated group in striatum (Fig. 6Bc) at 3 d and 7d after tMCAO. These data showed that SQ29548 dramatically promoted higher levels of SOD2 and catalase and decreased iNOS mRNA expression after ischemia/reperfusion.

### SQ29548 inhibited blood-brain barrier destruction in mice after tMCAO

**Figure 7:**
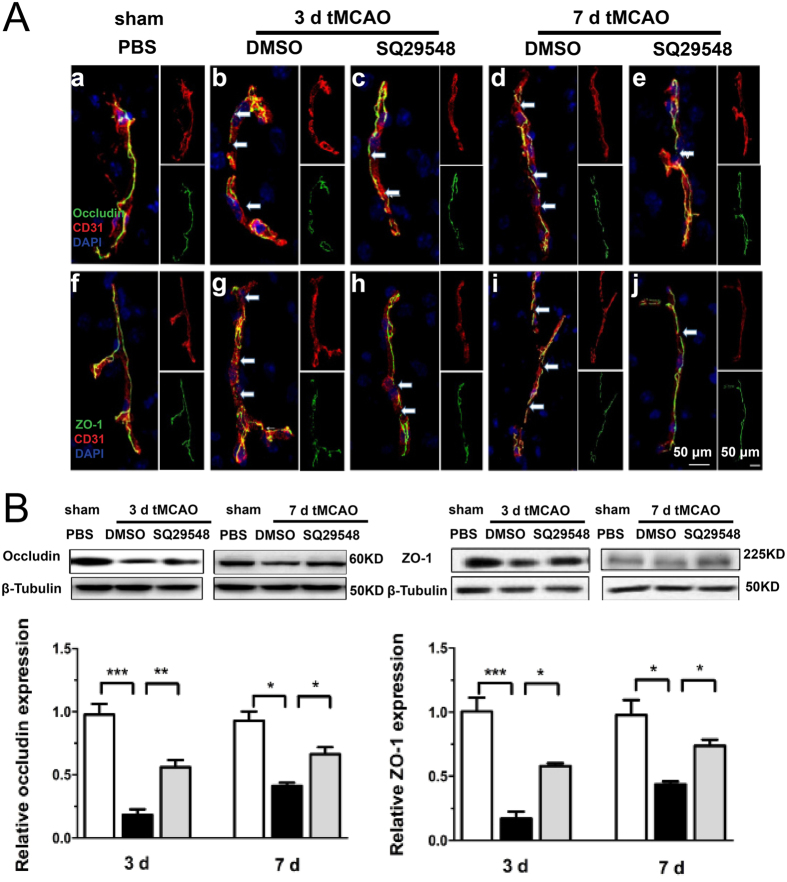
SQ29548 reduced blood-brain barrier destruction in mice after tMCAO injury. (**A**) Immunofluorescence double staining for CD31 with ZO-1 or occludin in the ipsilateral striatum of sham-PBS (n = 4), tMCAO-DMSO (n = 8) and tMCAO-SQ29548 (n = 8) groups at 3 d, 7 d following tMCAO. (**B**) Analysis of ZO-1 and occludin protein levels at 3 d, 7 d following tMCAO. Quantification of densitometric value of the protein bands normalized to the respective β-tubulin was also shown. Full-length blots/gels are presented in Supplementary Figure S2. The gels have been run under the same experimental conditions. Scale bar = 50 μm. Values are mean ± SEM. *P < 0.05, **P < 0.01, ***P < 0.001.

It has been reported that inflammatory mediators and oxidative stress response contribute to blood brain barrier (BBB) damage and the endothelial expression of adhesion molecules, thus we measured the BBB integrity by immunofluorescence staining and Western blotting. Confocal microscopic analysis indicated that the tight junction protein occludin and ZO-1 were continuously located along the endothelial cells (CD31 positive) of microvessels in the sham group (Fig. 7Aa,f). Microvessel walls showed significant disruption with discontinuous occludin staining along the margin in the DMSO-treated group at 3 d and 7 d after tMCAO (Fig. 7Ab,d, arrowhead), which could be rescued with SQ29548 treatment (Fig. 7Ac,e, arrowhead). Similar results were observed in ZO-1 staining (Fig. 7Ag,i vs. h,j, arrowhead). Consistent with the immunofluorescence double staining, Western blotting showed that there was less degradation of occludin and ZO-1 in the SQ29548-treated group than in the DMSO-treated group after tMCAO (Fig. 7B).

### SQ29548 protected against ischemic brain injury and improved neurological outcome

**Figure 8:**
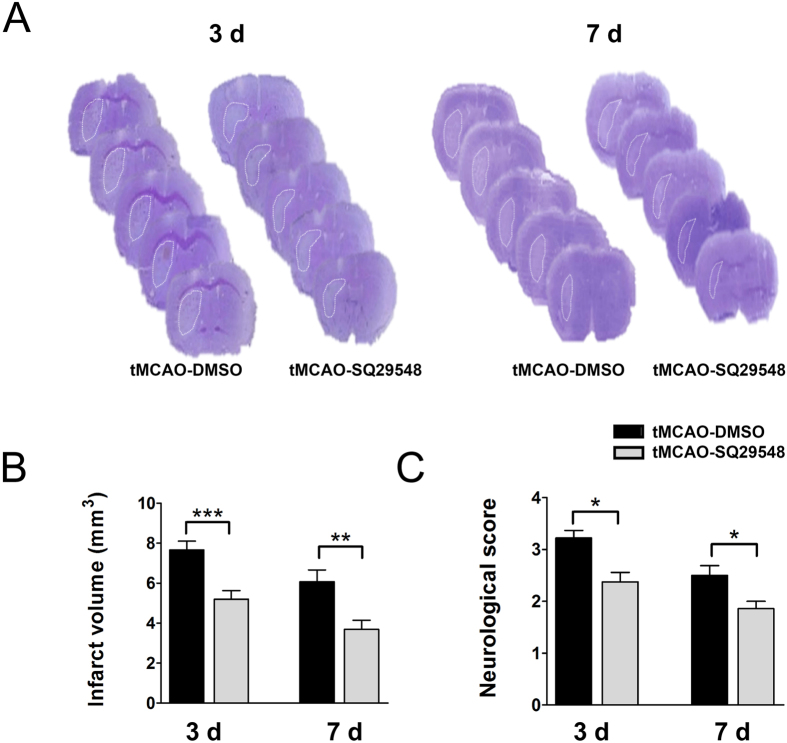
SQ29548 protects against tMCAO-induced cerebral injury. (**A**) Cresyl violet staining of brain sections from mice administered DMSO or SQ29548 at 3 d or 7 d after tMCAO (n = 4 each group). Dash line indicates infarct area. (**B**) Quantification of infarct volumes. (**C**) Neurological scores of DMSO- or SQ29548-treated mice at 3 d and 7 d after tMCAO (n = 8 each group). Values are the mean ± SEM. *P < 0.05, **P < 0.01, ***P < 0.001.

Inflammation, oxidative stress and activated microglia/macrophages are important factors that contributes to infarct area diffusion in ischemia/reperfusion injury^3^,^4^. Inhibition of microglia/macrophages activation and inflammation reaction after cerebral infarction can improve neurological outcome and ameliorate brain injury^6^,^7^,^24^. SQ29548 inhibited microglia/macrophages activation and reduced inflammation reaction and oxidative stress response, and then we tested the effect of SQ29548 in brain injury and neurological outcome after tMCAO. Our data showed the infarct volume was significantly smaller in the tMCAO SQ29548-treated mice than in the tMCAO DMSO-treated mice at 3 d (5.2 ± 0.4 mm^3^ vs. 7.7 ± 0.4 mm^3^, p < 0.001) and 7 d (3.7 ± 0.4 mm^3^ vs. 6.1 ± 0.6 mm^3^, p < 0.01) (Fig. 8A,B). Furthermore, SQ29548 effectively ameliorated neurological deficiency compared to the DMSO vehicle at 3 d (2.4 ± 0.2 vs. 3.2 ± 0.2, p < 0.05) and 7 d (1.8 ± 0.1 vs. 2.5 ± 0.2, p < 0.05) after tMCAO, as shown by neurological severity scores (mNSS) (Fig. 8C). These results suggested that post-treatment with SQ29548 reduced brain injury and neurological deficits in tMCAO mice.

## Discussion

In the present study, we showed that ischemic stroke was associated with an upregulation of TXA2R that was mostly co-localized to the activated microglia/macrophages in the mouse brain. We also observed that intracerebroventricular administration of TXA2R antagonist SQ29548 led to the inhibition of microglia/macrophages activation and enrichment including the classically activated M1 phenotype and the alternatively activated M2 phenotype after tMCAO. Furthermore, the effect of SQ29548 was largely associated with its anti-inflammatory and anti-oxidative stress property that contributed to integrity of BBB, the effective decrease in the infarct volume and improvement in the neurological outcome in tMCAO mice models.

Historically, TXA2R involvement in blood platelet function has received the greatest attention. However, it is now clear that TXA2R plays an important role among different organ systems. TXA2 is the ligand of TXA2R. The amount of TXA2 is increased dramatically in the brain after cerebral infarction injury^16^,^17^. TXA2 is unstable and rapidly degraded into an inactive form of TXB2, non-enzymatically. Due to the short half-life of TXA2 (t^1^/_2_ ~ 30 s)^25^, we did not test its concentration in our study. TXA2 exerts its action through the specific G protein-coupled TXA2R. Therefore, we detected the expression of TXA2R protein and mRNA in tMCAO mice. The data of our study showed TXA2R was upregulated in the ipsilateral striatum after tMCAO, and highly expressed in activated microglia/macrophages. Previous study reported activated microglia/macrophages cells are the principal source of brain derived TXA2^26^. Our previous study discovered that TXA2R agonist U46619 stimulated IL-1ß, IL-6, iNOS expression and IL-1ß, NO release in BV2 microglia and primary microglia. These data have suggested that stimulation of TXA2R signaling could be one of the underlying pathways for microglia activation^15^. We presumed that after cerebral infarction, TXA2 interact with TXA2R, which can activate the microglia/macrophages. A robust activation of microglia/macrophages may cause large amount of inflammatory cytokines secretion, leading to activation and enrichment of microglia/macrophages via autocrine or paracrine mechanisms and finally form a vicious circle. The vicious circle may also lead to the increased expression of TXA2R in microglia/macrophages.

Another key finding in this study was that TXA2R antagonist SQ29548 reduced the activation and enrichment of microglia/macrophages. Microglia, the resident immune cells, play an important role in host defense and immune surveillance under normal conditions^27^. Activated microglia/macrophages include two phenotypes, the classically activated M1 type that releases inflammatory mediators and the alternatively activated M2 type that releases anti-inflammatory cytokines^20^. Hu and coworker’s study showed that M2 microglia/macrophage was activated firstly after tMCAO^28^. The mRNA levels of M2 markers (Arg-1, Ym-1) increased at 24 h, peaked at 3 d after stroke and then began to decrease; while the mRNA levels of M1 markers (CD16, CD86) gradually increased over time from 3 d and remained elevated for at least 14 days after tMCAO^28^. In our study, for the DMSO-treated group, the expression of M1 genes including CD16 and CD86 at 7 d was higher than at 3 d after tMCAO. On the other hand, the expression of M2 hallmark Arg-1 and YM-1 at 3 d was higher than at 7 d after tMCAO. These data are in accordance with previous study. Our work also found that treatment of SQ29548 inhibited M2 markers (Arg-1, Ym-1) expression at 3 d and dampened the upregulation of M1 markers (CD16, CD86) at 7 d after tMCAO. The percentage of Iba-1^+^ microglia/macrophages was also obviously lower in tMCAO-SQ29548 group. This means that blockage of TXA2R after tMCAO inhibited resident microglia/macrophages to activate and enrich.

Activation of microglia/microphages contribute to the inflammatory responses and oxidative stress and the disruption of BBB after various neuronal disorders, such as cerebral infarction, Alzheimer’s disease and Parkinson’s disease^18^,^29^,^30^. Activated microglia/macrophages secrete various inflammatory factors in the ischemia/reperfusion process. The inflammatory response plays a key role in the damage processes of ischemic stroke. SQ29548 could ameliorate the activation and enrichment of microglia/microphages. So we observed the expression of pro-inflammatory cytokines and oxidative stress mediators. SQ29548 could down-regulate IL-1ß, TNF-α, IL-6, iNOS gene and also up-regulate antioxidant genes such as SOD2 and catalase. Furthermore, it could protect against the disruption of BBB and brain injury.

These results implicated the usefulness of TXA2R as a target for stroke therapy. Indeed, several previous investigations have indicated the use of TXA2R-based therapy for other diseases. For instance, improved outcome was observed after TXA2R antagonists treatment in mice subjected to spinal cord perfusion following experimental cord damage^31^. Another study has illustrated TXA2R antagonist SQ29548 provides protection against intestinal ischemia/reperfusion induced injury, evidenced by reducing inflammation^32^. Our study demonstrated thromboxane A2 receptor antagonist SQ29548 inhibited ischemia-induced inflammatory response and furthermore reduced microglia/macrophages activation, and ameliorated brain injury. Suppression of post-ischemic microglia/macrophages can protect against ischemic damage in the experimental tMCAO models. For example, therapies with adjudin^33^, minocycline^34^ inhibited a particular aspect of activated microglia/macrophages and protected against stroke-induced damage in an animal model of cerebral infarction. Our study added another small molecule to the list of neuro-protectants. However, some problems still need to be resolved. The dynamics of microglia/macrophages recruitment/activation in the damage and recovery phase of ischemic brain injury are complicated, and how TXA2R-mediated signaling functions in the process is not clear. Even though the expression level of TXA2R in astrocytes and oligodendrocytes is very low, it still remains to be investigated whether these cells participate in the SQ29548-mediated neuroprotection. In future studies, better characterization of activated microglia/macrophages from brain tissues of tMCAO model, longer observation window, and detailed examination on other cell types particularly astrocytes should be included. These further studies may be crucial for the elucidation of mechanisms in stroke therapy.

## Conclusions

The major discovery of the current study was that TXA2R in microglia/macrophages was upregulated in the ipsilateral striatum after tMCAO. The antagonist of TXA2R SQ29548 inhibited ischemic stroke-induced microglia/macrophages activation and enrichment, and exerted protective effects in ischemic stroke.

## Materials and Methods

### Reagents and animals

TXA2R antagonist (SQ29548) and DMSO (dimethyl sulfoxide) were purchased from Sigma Aldrich (St. Louis, MO, USA). ICR mice were purchased from Shanghai SLAG laboratory Animal Corporation (Shanghai, China).

### Animal experimental procedures

ICR mice weighting 25 to 30 g were anesthetized with ketamine (100 mg/kg) and xylazine (10 mg/kg, Sigma- Aldrich, San Louis, MO) intraperitoneally. Mice were randomly divided into the sham group, tMCAO group, sham + PBS-treated group, tMCAO + DMSO-treated group and tMCAO + SQ29548-treated group. Focal cerebral ischemia was produced by tMCAO as described previously^35^. In brief, beyond the common carotid artery, the left middle cerebral artery was occluded with a suture (Dermalon, 1741-11, Covidien, OH, USA) coated with silicone. Reperfusion was induced after 90 min by suture withdrawal. All procedures were performed under an operating microscope (Leica, Wetzlar, Germany). Regional cerebral blood occlusion and reperfusion were measured using a laser Doppler flowmetry (Moor Instruments, Devon, UK). Occluded mice had cerebral blood flow that was 20% of baseline and no hemorrhage were found. The sham mice underwent the same anesthesia and surgical procedures except for the suture insertion. The rectal temperature was controlled at 37.0 ± 0.5 °C during surgery by a temperature-regulated heating pad (RWD Life Science, Shenzhen, China). The procedures involving the animals were approved by the Ethical Committee of the Medical School of Shanghai Jiaotong University. The methods concerning animal surgery, care, euthanasia and protocol use were carried out in accordance with the approved guidelines and regulations of the National Institutes of Health for the care and use of laboratory animals.

### Intracerebroventricular administration of SQ29548

Due to an inability to cross the blood brain barrier, SQ29548 needs to be directly delivered into the central nervous system. Furthermore in the blood circulation, it could interact with the TXA2R of platelets and endothelial cells, and possibly influence the effect in the brain. Therefore we used intracerebroventricular injection rather than by intraperitoneal injection in our studies. The ICR mice received stereotaxic injection immediately after reperfusion of SQ29548 (10 mg/ml dissolved in DMSO at a dilution of 1:10 to 2.6 μmol/ml, 10 μl) or DMSO (DMSO dissolved in PBS at a dilution of 1:10, 10 μl) at a rate of 0.4 μl/min in the left lateral ventricle using a 10-μl Hamilton syringe (Hamilton, Bonaduz, Switzerland). The dose of SQ29548 was chosen based on the previous study^32^,^36^. The TXA2R protein level was further elevated at 24 h after tMCAO, therefore the second injection at the same dose was performed 24 h after the first injection. The injection coordinates were 1 mm lateral to the sagittal suture and 0.25 mm posterior to the coronal suture. The syringe was lowered into the brain 3 mm under the dura^37^. Five minutes after the completion of the injection, the needle was slowly withdrawn from the brain. All of the mice were sacrificed at either 3 d or 7 d after reperfusion.

### Infarct volume measurement

The mice from each group were sacrificed at either 3 d or 7 d after tMCAO. The brain tissue was cut into a series of 20-μm-thick sections from the beginning of the infarct area to the end. Brain cryosections were immersed in 0.1% cresyl violet for 30 min and then rinsed in distilled water for 15 min. The infarct volume was measured using the ImageJ program and the following formula: infarct area (mm^2^) = contralateral hemisphere area (mm^2^) − ipsilateral undamaged area (mm^2^). Infarct volume was calculated by the formula:


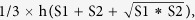


where S1 and S2 are the infarct areas of the two adjacent sections, and h is the distance between them. The total infarct volume was calculated as the sum of all infarct volumes from each pair of adjacent sections^38^.

### Neurological behavioral assessments

Neurobehavior was measured 3 d or 7 d after tMCAO by an experimenter who was blind to the experimental conditions. A modified Neurological Severity Score (mNSS) ranging from 0 to 14 was used, which included walking on the floor (range: 0 to 3), limb flexion (range: 0 to 3), beam balance tests (range: 0 to 6) and the absence of relex (range: 0 to 2)^39^. mNSS is a composite of motor, flexibility, reflex, and sensory balance tests. Every animal was tested three times, and the average score was recorded. A high score indicates more serious injury.

### Tissue preparation

In tMCAO group, mice were sacrificed at 6 h, 24 h, 3 d and 7 d after reperfusion and the brains were immediately removed. We collected the tissue of the ipsilateral striatum and cortex. In tMCAO DMSO-treated group and tMCAO SQ29548-treated group, for quantitative real-time PCR analysis, mice were sacrificed at 3 d or 7 d after reperfusion and the brains were immediately removed. We collected the tissue of the ipsilateral striatum. All tissues were stored at −80 °C.

### Immunostaining

Brain sections (20 μm in thickness) were fixed with 4% paraformaldehyde for 15 min and then incubated in PBS for 5 min. Slides were blocked for 30 min in 10% normal donkey serum and then incubated at 4 °C overnight with primary antibody. Primary antibodies used were rabbit anti-TXA2R (1:100 dilution, Santa Cruz Biotechnology, CA, USA); goat anti-Iba-1 (1:200 dilution, Abcam); mouse anti-GFAP (1:200 dilution, Millipore); rat anti-MBP (1:200 dilution, abcam); rat anti-CD16/32 antibody (1:200 dilution, BD Pharmingen, San Jose, CA, USA); goat anti-Arg-1 (1:100 dilution, Santa Cruz Biotechnology); rat anti-CD31 antibody (1:100 dilution, Life Technologies, CA, USA); rabbit anti-ZO-1 (1:100 dilution, Life Technologies, CA, USA); rabbit anti-Occludin (1:100 dilution, Invitrogen, Carlsbad, CA, USA). After washing three times with PBS, brain sections were incubated with corresponding donkey anti-rabbit, donkey anti-goat, or donkey anti-rat secondary antibodies conjugated to Alexa Fluor-488 and Alexa Fluor-594 fluorochrome (1:400 dilution, Life Technologies). 4,6-diamidino-2-phenylindole (DAPI) (1:500 dilution, Beyotime Institute of Biotechnology, China) was used to stain the nuclei. The brain sections were viewed using a confocal microscope (Leica, Solms, Germany).

### Quantitative polymerase chain reaction

Total RNA samples from the brain tissue were isolated using TRIzol Reagent (Life Technologies) and were reversely transcribed to cDNA using the PrimeScript RT reagent kit (TaKaRa). qPCR was performed using a SYBR Green kit (TaKaRa) according to the manufacturer’s instructions. qPCR was performed using the following conditions: denaturing at 95 °C for 10 s, followed by 40 cycles of 95 °C for 5 s and 60 °C for 30 s. The data were analyzed by the comparative threshold cycle (Ct) method, and the results were expressed as fold difference normalized to ribosomal phosphoprotein P0 (Rplp0). The primer sequences were as follows: TXA2R (F: ATCTCCCATCTTGCCATAGTCC, R: CCGATGATCCTTGGAGCCTAAAG); CD16 (F: TTTGGACACCCAGATGTTTCAG, R:GTCTTCCTTGAGCACCTGGATC); CD86 (F: TTGTGTGTGTTCTGGAAACGGAG, R: AACTTAGAGGCTGTGTTGCTGGG); Arg-1 (F: GAACACGGCAGTGGCTTTAAC, R: TGCTTAGCTCTGTCTGCTTTGC); YM-1 (F: GGAGTAGAGACCATGGCACTGAAC, R:GACTTGCGTGACTATGAAGCATTG); IL-1ß (F: GCAACTGTTCCTGAACTCAACT, R: ATCTTTTGGGGCGTCAACT); IL-6 (F: TAGTCCTTCCTACCCCAATTTCC, R:TTGGTCCTTAGCCACTCCTTC); TNF-α (F: CCCTCACACTCAGATCATCTTCT, R:GCTACGACGTGGGCTACAG); IL-10 (F: GCTCCAAGACCAAGGTGTCTACAA, R:CCGTTAGCTAAGATCCCTGGATCA); Catalase (F:ACGCAATTCACACCTACACG, R:TCCAGCGTTGATTACAGGTG); SOD2 (F:GCGGTCTAAACCTCAAT, R:TAGGGCTCAGGTTTGTCCAG); iNOS (F: ATGTCCGAAGCAAACATCAC, R:TAATGTCCAGGAAGTAGGTG); Rplp0 (F: AGATTCGGGATATGCTGTTGGC, R: TCGGGTCCTAGACCAGTGTTC).

### Western blotting

Protein samples were prepared by homogenizing ipsilateral striatum and cortex tissues in standard lysis buffer. The protein concentration was measured using a BCA kit (Thermo Scientific). Equal amount of protein was separated by 10% or 6% (for ZO-1) SDS-PAGE and then transferred to nitrocellulose membranes (300 mA for 70 min). The membranes were blocked with 5% BSA for 1 h at room temperature and incubated with primary antibodies overnight at 4 °C. Membranes were washed three times with TBST and then incubated with secondary antibody for 1 h at room temperature. The specific bands were visualized with enhanced chemiluminescence (Thermo Scientific). The results were measured with an imaging system (Bio-Rad). The primary antibodies used were: TXA2R (1:500, Santa Cruz)^11^,^15^; ZO-1 (1:1000, Invitrogen)^33^; Occludin (1:1000, Invitrogen)^33^; β-tubulin (1:2000, Sigma); and actin (1:1000, Santa Cruz).

### Statistical analysis

All statistical analyses were performed using GraphPad Prism V5.0. For comparison between the two groups, statistical significance was determined through a Student’s t test. For comparison among multiple groups, statistical significance was evaluated using one-way ANOVA followed by a Student-Newman-Keuls test. Data were expressed as the mean ± SEM. P values < 0.05 were considered statistically significant.

## Additional Information

**How to cite this article**: Yan, A. *et al*. Thromboxane A2 receptor antagonist SQ29548 reduces ischemic stroke-induced microglia/macrophages activation and enrichment, and ameliorates brain injury. *Sci. Rep.* **6**, 35885; doi: 10.1038/srep35885 (2016).

## Supplementary Material

Supplementary Information
