# Supplementary material for: Thromboxane A2 receptor antagonist SQ29548 reduces ischemic stroke-induced microglia/macrophages activation and enrichment, and ameliorates brain injury
Source: Sci Rep. 2016 Oct 24;6:35885. doi: 10.1038/srep35885 (PMC5075919; doi:10.1038/srep35885)
Supplement: Supplementary Information [file srep35885-s1.pdf]

Thromboxane A2 receptor antagonist SQ29548 reduces ischemic stroke-induced microglia/macrophages activation and enrichment, and ameliorates brain injury

Aijuan Yan[1#], Tingting Zhang[1#], Xiao Yang[1], Jiaxiang Shao[1], Ningzhen Fu[2], Fanxia Shen[1], Yi Fu [1*], Weiliang Xia [1*]

TXA2R protein

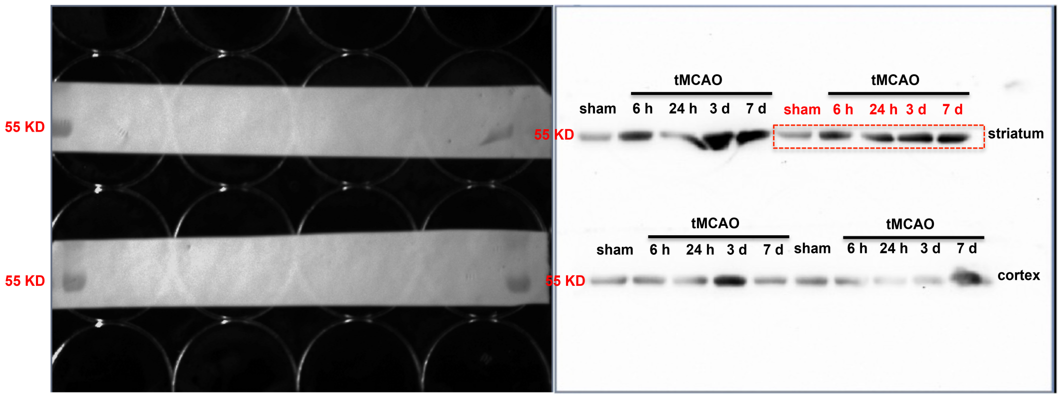

Figure S1 The full-length gels and blots of TXA2R protein expression in the ipsilateral striatum and cortex after tMCAO (n=4 each time point).

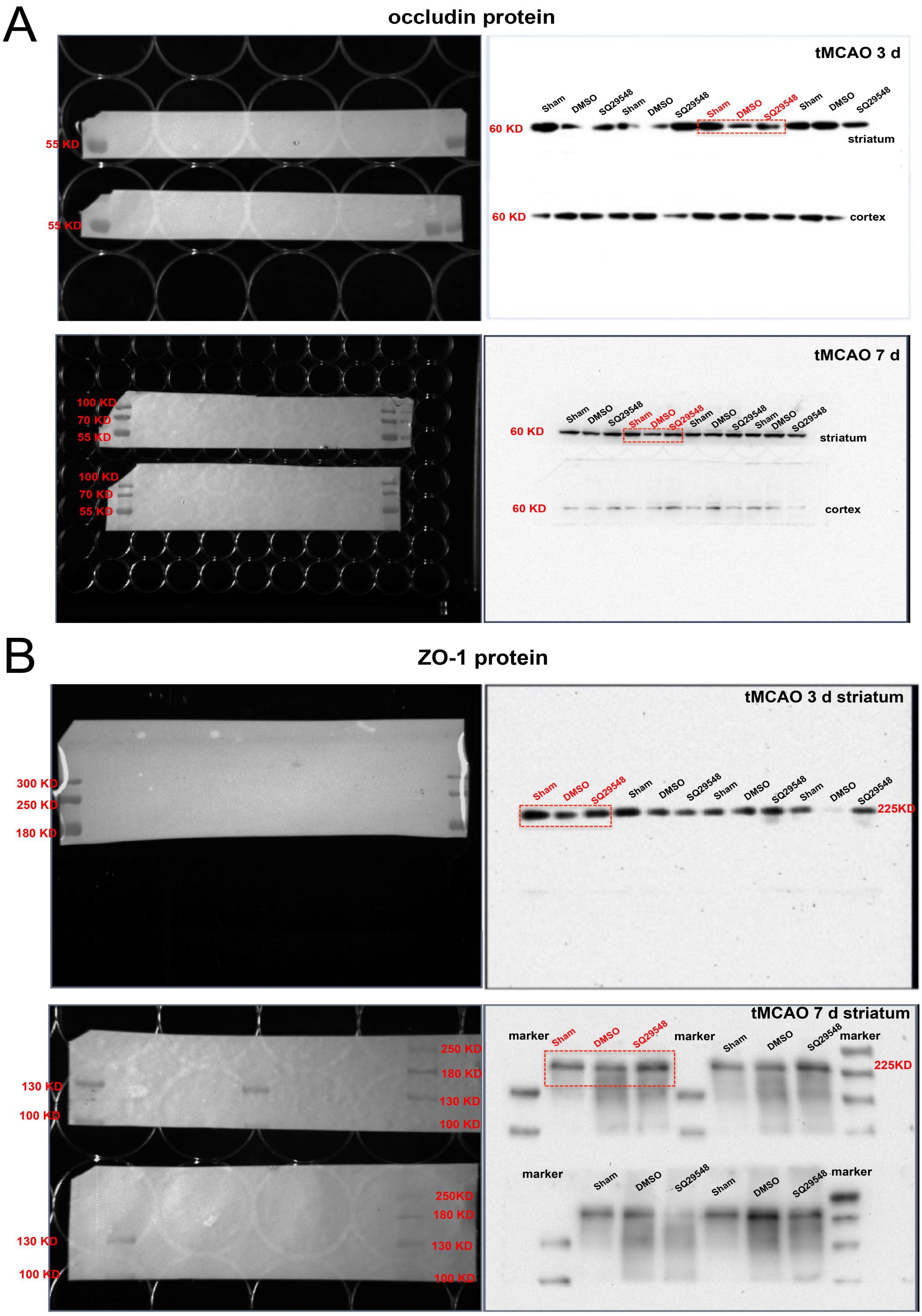

Figure S2 The full-length gels and blots of occludin and ZO-1 protein expression in the ipsilateral semisphere at 3 d and 7 d after tMCAO(n=4).

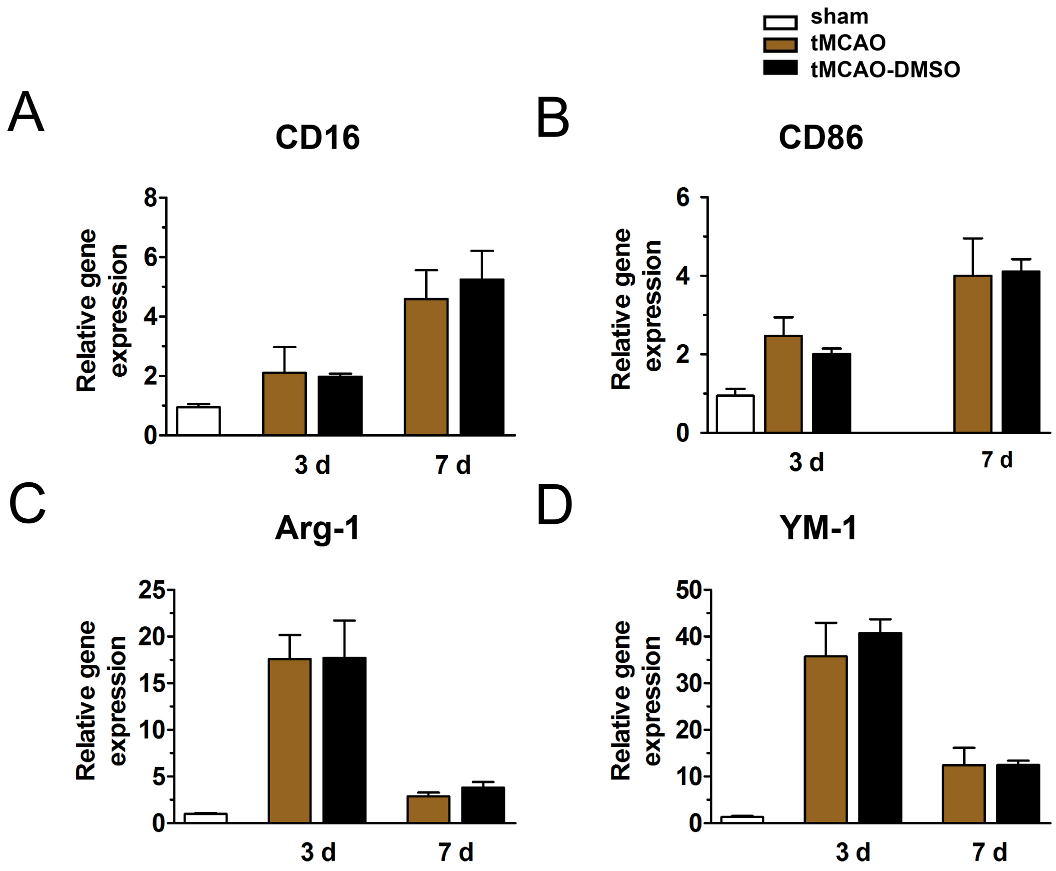

Figure S3. Evaluation of the effect of DMSO on microglia/macrophages activation after tMCAO. mRNA levels of M1 markers CD16 (A) and CD86 (B) and M2 markers Arg-1(C) and Ym-1(D) in the ipsilateral striatum of sham (n=4), tMCAO (n=8) and tMCAO-DMSO (n=8) groups at 3 d, 7 d following tMCAO was analyzed by qPCR. Values are the mean ± SEM.

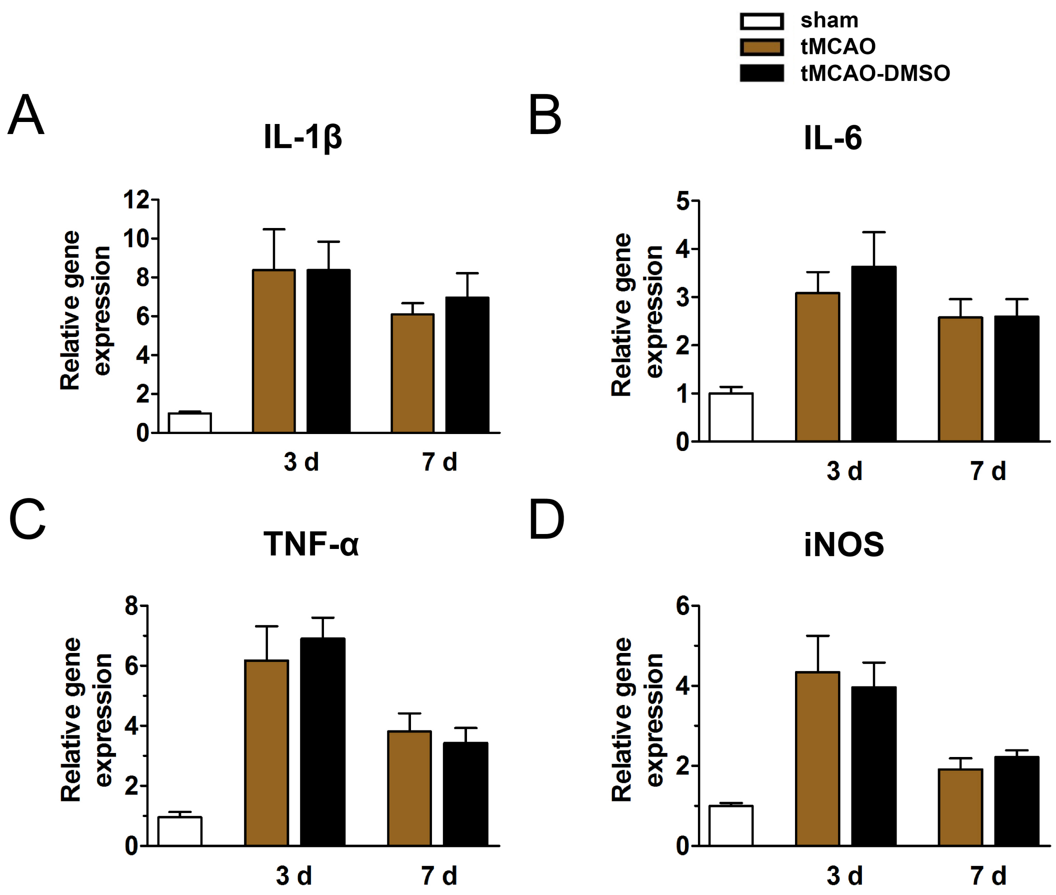

Figure S4. Evaluation of the effect of DMSO on the expression of inflammatory cytokines after tMCAO injury. mRNA levels of IL-1ß (A), IL-6 (B), TNF-α (C), and iNOS (D) were measured in the striatum of sham (n=4), tMCAO (n=8) and tMCAO-DMSO (n=8) groups at 3 d and 7 d following surgery. Values are the mean ± SEM.

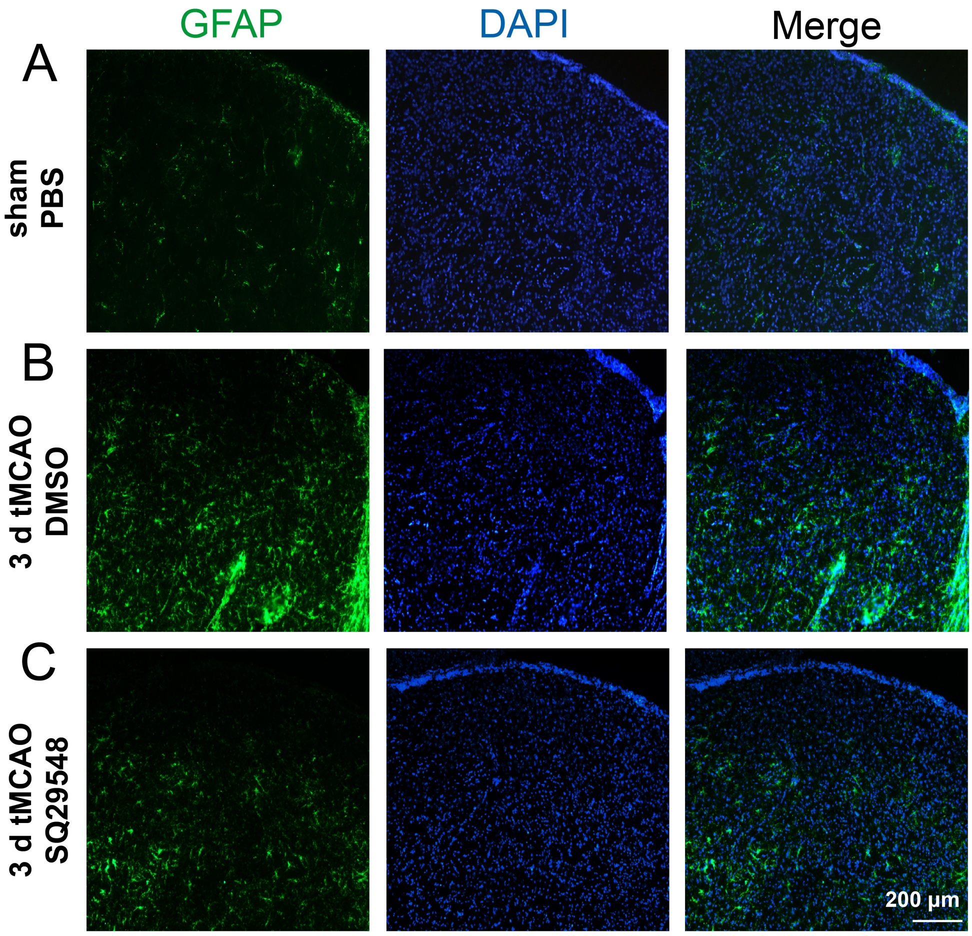

Figure S5. SQ29548 inhibits astrocytes activation after tMCAO injury. (A, B, C) Immunofluorescence staining for GFAP (green) in the ipsilateral striatum of sham-PBS (n=4), tMCAO-DMSO (n=4) and tMCAO-SQ29548 (n=4) groups at 3 d following tMCAO. Scale bar=200 µm.

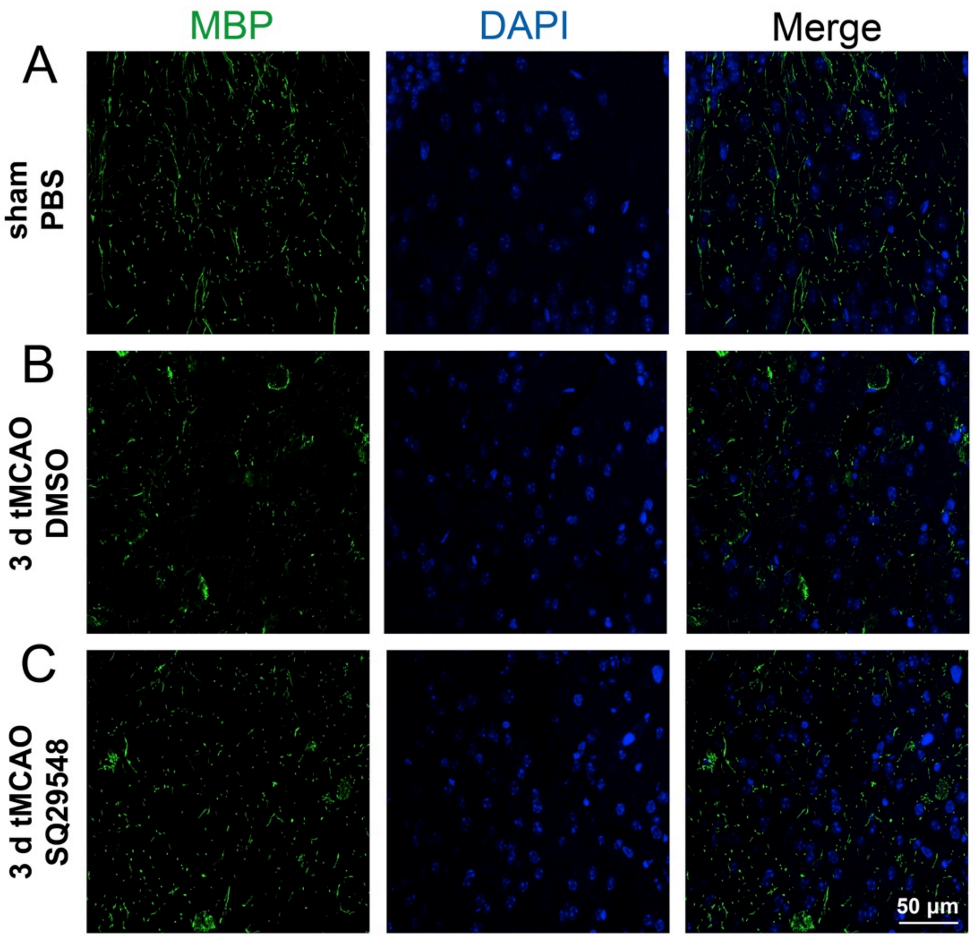

Figure S6. SQ29548 increased the expression of MBP after tMCAO injury. (A, B, C) Immunofluorescence staining for MBP (green) in the inner boundary of the infarct striatum of sham-PBS (n=4), tMCAO-DMSO (n=4) and tMCAO-SQ29548 (n=4) groups at 3 d following tMCAO. Scale bar=50 μm.
